# AI in the Classroom: Observing Preclinical Students' Use of ChatGPT During Case‐Based Learning at a UK Medical School

**DOI:** 10.1111/tct.70360

**Published:** 2026-02-13

**Authors:** A. W. R. Taylor, F. Y. Patel, E. K. Smyth, S. Gay, T. Bird

**Affiliations:** ^1^ University of Leicester Leicester UK

**Keywords:** active learning, AI chatbots, artificial intelligence in education, case‐based learning, undergraduate medical education

## Abstract

**Background:**

This article explores how preclinical students in a UK medical school utilise ChatGPT during their case‐based learning (CBL) curriculum.

**Materials and Methods:**

Focused ethnography was used to study 42 medical students and three clinical sciences students as they undertook seven CBL sessions over 4 weeks. In situ observations, screenshots of ChatGPT conversations and focus group data were collected and analysed using reflexive thematic analysis.

**Results:**

ChatGPT was used to automate concept retrieval, problem‐solving and applying theory to clinical context. This occurred because students were motivated by the efficient completion of workbook questions, which could be achieved by entering them into ChatGPT. Collaborative groups were less likely to automate cognitive effort because they placed greater value on being engaged during learning and perceived socially constructing answers as more efficient than ChatGPT use. Although ChatGPT sometimes gave partial or false‐but‐plausible answers, students were rarely observed cross‐checking it.

**Conclusions:**

AI chatbot use during conventional curricular activities can result in students automating cognitive effort. This is more likely during tasks scaffolded by guiding materials and when students are motivated by external pressures rather than a desire for engaging learning. Educators should specify how and when AI chatbots should be used, promote autonomy and collaboration in student cultures and teach students about the value of cognitive effort to learning.

## 1 Background

Medical students are increasingly using AI chatbots like ChatGPT to learn [1, 2], including during curricular activities [2]. There is agreement that chatbots can benefit students by offering immediate answers and feedback, promoting self‐regulated learning [3, 4, 5]. Accounts of ‘hallucination’ are also common, where false‐but‐plausible answers are generated that students are at risk of absorbing. Though some medical students share concerns over chatbot accuracy [6], they mostly perceive ChatGPT positively, see its benefit as a learning aid [3] and believe it improves critical thinking skills [7].

There are inconsistencies in the literature about how chatbots affect higher‐order thinking [3, 4, 5, 8]. An umbrella review found that students used chatbots to evaluate counterarguments in some studies, but critical evaluation was reduced in others. Similarly, a systematic review [5] examining medical education reported findings that chatbots might reduce critical thinking, but also that they enhance critical thinking by facilitating two‐way dialogue and the formation of hypotheses by students. While these reviews included non‐empirical studies, a systematic review of only experimental research found that ChatGPT use enhanced higher‐order thinking and reduced cognitive effort, suggesting empirical support for positive effects [9]. Case‐based learning (CBL) relies on students applying theory to clinical cases through self‐regulated learning, collaboration and critical thinking [10], so ChatGPT use is likely to influence its effectiveness [11].

There is little empirical data on the impact of AI chatbots on medical education [4], including qualitative data [12], with conclusions based mostly on editorials and studies testing chatbots against exams [4, 5, 12]. Also, most experimental research assessed interventions designed for chatbot use [9], not the impact it has on existing curricular activities. Authors call for research exploring the impact of chatbots on existing educational content [4], student perspectives of ChatGPT [5] and effects on higher‐order thinking [8]. No ethnographic studies on medical student interactions with ChatGPT were found.

As well as gathering in situ student perspectives, ethnographic data would provide insight into how they *actually use* ChatGPT during curricular active learning activities. If students use ChatGPT to avoid problem‐solving, why? If it promotes critical thinking, how? To explore why and how health professions students interact with AI chatbots in existing medical curricula, this study took AI research into the classroom to answer the question, ‘How do preclinical students in a UK medical school use ChatGPT in case‐based group work?’.

## 2 Materials and Methods

### 2.1 Study Design and Ethical Approval

This study adopted a critical realist paradigm, acknowledging an underlying reality accessible only through interpretation. Focused ethnography was used to enable data‐rich fieldwork exploring an aspect of a culture, namely, AI chatbot use during group work, over a short timeframe [13]. Therefore, data reflected observations and in situ reflections, so post hoc student interpretations were minimised. Reflexive thematic analysis (RTA) was chosen as it enables both descriptive and interpretive accounts, integrating researchers' observations with students' perspectives [14, 15]. Due to the novelty of the research area, there was minimal information to support the selection of a relevant theoretical lens. Instead, themes were generated inductively, with theoretical links drawn post‐analysis in the discussion [16]. To minimise harm to learning, participants were asked to watch a video about optimal AI chatbot use. Ethical approval was received from the Medicine and Biological Sciences Research Ethics Committee at the University of Leicester (43564‐at631‐Is:medicine).

### 2.2 Reflexivity

The researchers who collected and analysed data were clinical doctors, educators and graduates of the CBL curriculum studied, with two also serving as participants' tutors. This insider perspective is recognised in RTA as a strength, enabling richer interpretations of data [16]. Awareness of how subjectivity shaped the research process was maintained by keeping reflexive journals. Before data collection, the lead researcher assumed that ChatGPT would promote active learning via elaboration, but the dual researcher‐tutors assumed that students would use it for superficial answers.

### 2.3 Setting and Participants

Second‐year preclinical undergraduates at the University of Leicester were sampled purposively; students in the researcher‐tutors' usual CBL groups were invited. They received an information leaflet and consent form via email (Supporting Information S1: Appendix A), and the study was explained in person. As part of their usual MB ChB curriculum, students attended a lecture, then split into rooms with four groups of eight or fewer to do a 2‐h CBL session using iPads. They used digital workbooks containing clinical cases and scaffolding questions that facilitated consolidation of lecture content via recall, elaboration via inquiry using lecture slides and the internet, and critical thinking via collaborative problem‐solving and application of theory to cases. A medically trained tutor provided scaffolding and feedback. Once all questions had been completed and reviewed by the tutor, students could leave.

### 2.4 Data Collection

**FIGURE 1:**
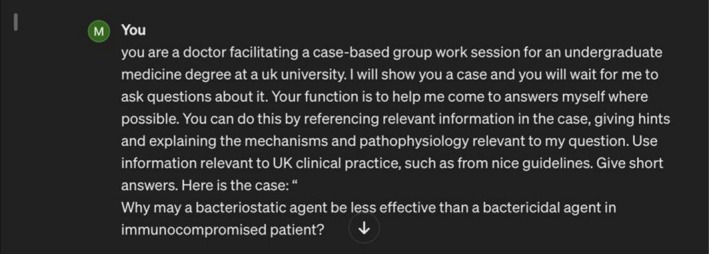
Example of a student using the ChatGPT ‘Persona’ instructions.

Between January and March 2024, seven CBL sessions were observed across two modules: three in Neurology and four in Pharmacology, enabling data triangulation. The lead researcher observed all sessions, while the researcher‐tutors observed their module only, so two researchers observed each session, enabling researcher triangulation. Field notes were written using free‐text entries supported by optional prompts on a template (Supporting Information S1: Appendix B), during or immediately after sessions. Students also submitted screenshots of ChatGPT conversations (Figure 1). After the final session of each module, participants were split into two semi‐structured focus groups (Supporting Information S1: Appendix C), one by each researcher present, lasting 20–60 min. Collecting three data types enabled methodological triangulation. The final materials were four focus group transcripts, 30 field note entries covering 30 h of CBL and 114 screenshots.

### 2.5 Data Analysis

To enable researchers' insider perspectives to shape theme generation, they independently analysed their own field notes and focus groups and divided screenshots from the modules they observed. Focus groups were transcribed, and researchers read and listened to all the data to familiarise themselves with it and generate initial impressions. Segments relevant to the research question were highlighted and reduced to semantic and latent codes, and patterns perceived by researchers became themes and subthemes. Once themes were finalised separately, the researchers met to discuss findings and how subjectivity may have shaped them. Similar themes were synthesised, and the most meaningful were refined into the final themes. Last, the researchers met with the project supervisor to make final adjustments. Member checking and external intercoders were not used to avoid moving themes away from the situated perspective of the researchers [15].

## 3 Results

Out of 56 students, 45 participated in the study (26 in Neurology, 19 in Pharmacology); of these, 17 were male and 28 were female, all aged 18–25. 41 participants were medical students, while three studied clinical sciences. 35 participants joined focus groups. ChatGPT use was voluntary, though students were encouraged to try Bing Copilot in the first session. Most preferred ChatGPT‐3.5 due to prior familiarity; some had used ChatGPT in CBL before, while others used it for the first time in this study. None watched the educational video about ChatGPT. Codes following exemplar quotes in the tables (e.g., *(R3‐P‐FG‐P16d)*) refer to *researcher number, module (Pharmacology (P) or Neurology (N)), data type (focus group (FG) or field notes (FN)), and participant code*.

### 3.1 Students Value Resource Efficiency, but Greater Resource Efficiency Can Reduce Critical Thinking

As shown in Table 1, learners' motivation to efficiently answer workbook questions was incongruent with the cognitive effort intended by the pedagogical approach. When this motivation dominated, ChatGPT could be used to automate the question‐answering process, providing an ‘easy way out’ (P42h).

**TABLE 1:** Quotes supporting the theme, ‘Students value resource efficiency, but greater resource efficiency can reduce critical thinking’.

Theme: Students value resource efficiency, but greater resource efficiency can reduce critical thinking	
Subthemes	Quotes
Students were more concerned with efficiency than any other resource characteristic	‘Some stated that they found [ChatGPT] to be more useful than Google, as you did not need to look at multiple resources to find your answer.’ (R3‐P‐FN) ‘P37g said they do not tend to read around the topic in group work because there are too many questions, and they just want to get through them.’ (R1‐N‐FN‐P37g) ‘I think [ChatGPT's] just fast, isn't it? I think the main issue is […] lack of trust. […] I'm not too fussed about that. I think majority of the time, it's, I'm assuming it's gonna get it right’. (R1‐N‐FG‐P36g)
ChatGPT can be used to find an answer without using critical thinking	P01a: ‘If we don't understand things, [we] just put it in ChatGPT instead of trying to figure out [the answer].’ R1: ‘Is that a good thing or a bad thing, do you think?’ P01a: ‘I think it's a bad thing.’ R1: ‘Do you think you are gonna carry on doing it?’ P03a: ‘I'll try not to but … (laughing)’. (R1‐P‐FG‐P01a‐P03a) ‘It could make it so easy to use, you are not thinking enough about it if you just skip that slide’. (R3‐P‐FG‐P14c)

#### 3.1.1 Students Were More Concerned With Efficiency Than With Any Other Resource Characteristic

Lecture slides were favoured because they often provided the fastest answer. ChatGPT was judged slow by some, especially in collaborative cultures, but often perceived as faster than searching online. Although depth and breadth were occasionally valued, the pressure of completing the workbook and covering the whole syllabus led to the prioritisation of speed and specificity.

#### 3.1.2 ChatGPT Can Be Used to Find an Answer Without Using Critical Thinking

Some students reported disengagement when using ChatGPT, saying they ‘stop thinking’ (P19d). Screenshots showed frequent submission of superficial queries or direct copy‐pasting of workbook questions (Figure 1). Outputs could then be pasted back into the workbook. This bypassed retrieval, elaboration, application of theory to cases and group problem‐solving. Adding case details exacerbated this as ChatGPT's answers were even more aligned with the intended answers. Some students recognised this was suboptimal, and of these, some stopped using ChatGPT, but others continued, prioritising efficiency over active learning.

### 3.2 Interactions With ChatGPT Were Superficial and Short

As shown in Table 2, students perceived ChatGPT as an answer finder, so prompting was focused on shortening outputs and improving relevance, and ChatGPT was not used for higher‐order processes like analysis or elaboration. A researcher‐designed prompt intended to encourage scaffolding conversations was rarely used, likely because it conflicted with students' conceptualisation of ChatGPT's function.

**TABLE 2:** Quotes supporting the theme, ‘Interactions with ChatGPT were superficial and short’.

Theme: Interactions with ChatGPT were superficial and short	
Subthemes	Quotes
ChatGPT is used like Google, not like a tutor	‘I just thought it was like Google, like you trust Google. You ask it a question and some words appear.’ (R3‐P‐FG‐P16d)
ChatGPT answers are perceived as long	‘And then you cannot really like ask ChatGPT to stop typing and answer you that. You have to wait.’ (R1‐N‐FG‐P34g)
Answers are not tailored to students' specific context	‘[ChatGPT gives] all the information at once in a way that could be the same for everyone.’ (R1‐N‐FG‐P38g)
Prompting increases relevance to the session	‘… we said, “from a UK point of view”. Like, putting that prompt in gave you a bit of a different answer compared to its natural US point of view’. (R1‐P‐FG‐P01a)

#### 3.2.1 ChatGPT Is Used Like Google, Not Like a Tutor

Many students perceived ChatGPT as like Google, resulting in short, one‐way ‘question‐answer’ interactions. It was used to find answers to workbook questions rather than dialogic exploratory questioning.

#### 3.2.2 ChatGPT Answers Are Perceived as Too Long

Students frequently described responses as overly lengthy and too slow. Many students inputted prompts like ‘be brief’, emphasising the focus on solutions over deeper learning.

#### 3.2.3 Answers Are Not Tailored to Students' Specific Context

Students often received patient‐focused answers. Because they seldom provided case details, likely because it would take longer, ChatGPT defaulted to generic responses or advised consulting a doctor.

#### 3.2.4 Prompting Increases Relevance to the Session

Students improved relevance with prompts, such as specifying the learner level. Observing this, researchers designed a context‐setting prompt that instructed ChatGPT to act as a tutor and avoid giving direct answers (Figure 1). It improved relevance, but most did not use it, likely because it took longer, reducing superficial efficiency. Even when used, students could still elicit direct answers from ChatGPT.

### 3.3 ChatGPT Use Depends on Group Dynamics

As shown in Table 3, collaboration strongly shaped how and when ChatGPT was used. Less collaborative cultures tended to use it earlier, more often and more passively. Highly collaborative cultures tended to avoid it or use it more critically.

**TABLE 3:** Quotes supporting the theme, ‘ChatGPT use depends on group dynamics’.

Theme: ChatGPT use depends on group dynamics	
Subthemes	Quotes
Groups with a weaker collaborative culture use ChatGPT sooner and more passively	‘This ends up not being a discussion [in less interactive groups] so much as students sharing GPT's answer and writing it down. This means the answer has not resulted in much collaboration‐ the delivery of answers is didactic.’ (R1‐P‐FN) ‘One of the student groups, even prior to the research, did not generally work together […] This group utilised ChatGPT more than any other group as they were not interacting […] and thus lacked that peer support.’ (R2‐N‐FN)
Groups with a stronger collaborative culture use ChatGPT less frequently and more collectively	‘I prefer the conversational sort of aspect over like Google or ChatGPT because I can like ask you directly without needing to rephrase the question all over again’. (R2‐N‐FG‐P45h) ‘One person ChatGPT'd it, and then we'll chat […] if the ChatGPT answer is unclear, can like work out through that and that will encourage discussion’. (R1‐P‐FG‐P04b) ‘It was straight away. Let's ask ChatGPT. […] [we would usually] like, discuss it more? […] I don't think I'd like to keep using it in group work […] I don't like that we stop thinking.’ (R3‐P‐FG‐P16d)

#### 3.3.1 Groups With a Weaker Collaborative Culture Use ChatGPT Sooner and More Passively

Less interactive groups were more likely to use ChatGPT for passive question‐answering and to use it before or instead of group discussion. Outputs were often accepted without group analysis or cross‐checking.

#### 3.3.2 Groups With a Stronger Collaborative Culture Use ChatGPT Less Frequently and More Collectively

Groups with stronger collaborative norms were more motivated to engage in active learning, though efficiency was still important. They typically attempted recall and problem‐solving collectively before or instead of consulting ChatGPT, and they often debated its outputs. Perceptions that ChatGPT reduced thinking came mostly from these groups, likely because their active, dialogic approach provided contrast. Some felt ChatGPT use diminished interaction, and many such groups did not use it at all during CBL.

### 3.4 When ChatGPT Is Wrong, Students May Not Notice

As shown in Table 4, students knew that ChatGPT could make mistakes, yet cross‐checking was infrequently observed. ChatGPT was used most when students were stuck or lacked other resources. In such situations, errors are harder to detect, increasing the risk of learning false information. Table 4 shows exemplar quotes for this theme.

**TABLE 4:** Quotes supporting the theme, ‘When ChatGPT is wrong, students may not notice’.

Theme: When ChatGPT is wrong, students may not notice	
Subthemes	Quotes
ChatGPT can give wrong or incomplete information	‘You have to take what ChatGPT says with a pinch of salt because a lot of the time it makes mistakes, but I think it's better to have the answers’. (R2‐N‐FG‐P42h)
Students rarely cross‐reference ChatGPT outside of verifying with the tutor.	‘I stopped checking on [ChatGPT]. Because […] if it's matched with the slides pretty much every time, then I'm going to assume that it does do that every time.’ (R3‐C‐FG‐P08b)
ChatGPT is used most on topics where students have the least knowledge and fewer resources	‘[You use ChatGPT] When you get like, really stuck. Like, if you are by yourself, like revising, you have got to the point where you have checked all the slides, and you still can't find the answer.’ (R2‐N‐FG‐P45h) ‘Students stated that they were using ChatGPT on the questions that they were not sure on at all’. (R3‐P‐FN)

#### 3.4.1 ChatGPT Can Give Wrong or Incomplete Information

**FIGURE 2:**
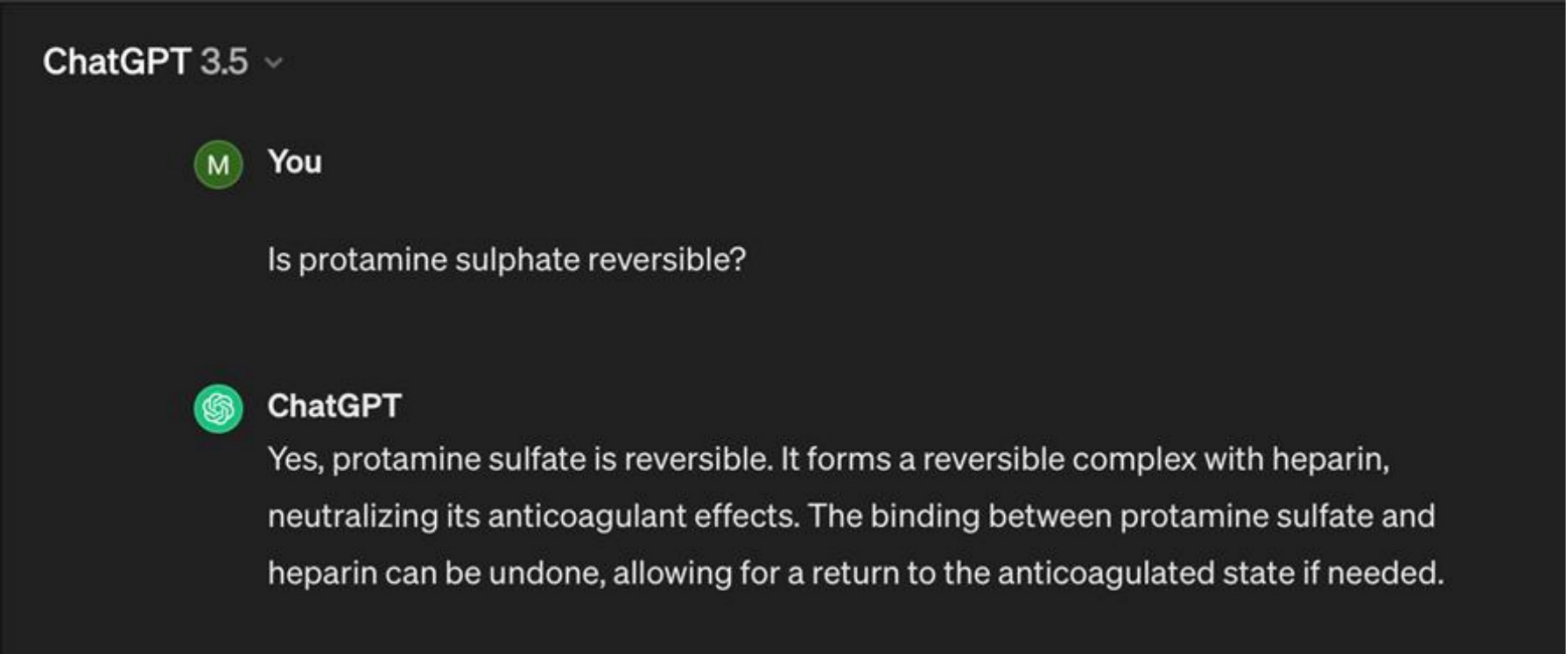
Example of ChatGPT giving incorrect information.

On one occasion, ChatGPT incorrectly stated that protamine binding was reversible (Figure 2). This statement was plausible and only noticed because the correct mechanism was discussed in the lecture. More often, ChatGPT gave incomplete responses.

#### 3.4.2 Students Rarely Cross‐Reference ChatGPT Outside of Verifying With the Tutor

ChatGPT was trusted enough that many students did not cross‐check outputs with other sources. Confidence in ChatGPT being right ‘pretty much every time’ (P08b), and in their own ability to spot errors, reinforced this behaviour. The primary form of student‐led cross‐checking was debate of ChatGPT's outputs in collaborative cultures, while tutor feedback provided a safety net, as all answers were reviewed.

#### 3.4.3 ChatGPT Is Used Most on Topics Where Students Have the Least Knowledge and Fewer Resources

Across all groups, ChatGPT was used mainly when students were stuck rather than to check understanding, making errors less likely to be noticed. Many students, including those in collaborative cultures, also relied on it during self‐study in place of peers or tutors, increasing the risk of errors being absorbed.

## 4 Discussion

### 4.1 Study Overview

The study explored how health‐profession students used AI chatbots during curricular CBL and identified factors shaping their impact on active learning. Students' use of ChatGPT depended on their motivations. By imposing an external learning structure, workbooks reduced student autonomy, fostering a focus on efficient answer‐finding and supplying text for ChatGPT inputs, enabling passive answer generation. Students' motivations were also shaped by group dynamics: In less collaborative groups, efficient answer‐finding dominated, while in collaborative cultures, a stronger drive for cognitive challenge reduced or eliminated passive ChatGPT use. More students used it at home than in group work to replace human support.

### 4.2 Comparison to Existing Theory and Research

Autonomy, relatedness and competence underpin the internal drive to learn for personal satisfaction, known as intrinsic motivation [17], and these factors influenced students' ChatGPT use in the present study. Structured questioning limited autonomy, which reduced intrinsic motivation and fostered a focus on efficiently finding answers. However, in collaborative cultures, socially constructing solutions enhanced perceived relatedness and competence, strengthening students' intrinsic motivation for engaging, challenging learning, leading to less passive chatbot use. This suggests that the degree to which a learning culture supports autonomy, relatedness and competence could predict how students are likely to interact with chatbots.

The present findings align with the literature on the importance of human tutors [3, 4, 5], ChatGPT's usefulness for information acquisition and the risk of hallucination, but add that students' awareness of errors may not motivate cross‐checking. Additionally, the findings provide insight into inconsistencies in the literature regarding chatbots' impact on higher‐order thinking [3, 4, 5]. Differing conclusions resulted from differences in the pedagogical frameworks and student motivations within the authors' contexts. For example, enhanced critical thinking was reported in studies of chatbot use during open inquiry [18], which involves greater student autonomy, while research on CBL reported reduced critical thinking [19].

When revising concepts, students often prefer passive study [20], but strategies that demand mental effort, like retrieval practice and elaboration, are significantly more effective at promoting long‐term retention [21, 22, 23]. Therefore, it is likely that in the present study, using ChatGPT to avoid mental effort led to more subsequent forgetting. A systematic review also found that ChatGPT reduced mental effort [9], and while this was attributed to reduced cognitive load, it may also reflect reduced learner engagement in retrieval and elaboration. This might explain why ChatGPT was less effective when studied over longer time periods [9]: Its efficiency improved immediate student performance, but reduced cognitive effort and accelerated forgetting [22]. Consequently, even in learning methods with high autonomy, ChatGPT may undermine retention if students lack knowledge of effective strategies, like retrieval practice, or the intrinsic motivation to use them.

### 4.3 Implications for Practice, Policy and Future Research

Clinical teachers can anticipate the impact of chatbots by considering the learning approaches and students' motivations in their context and minimise passive use by manipulating these factors. Autonomy could be promoted through exercises where students control learning, such as students creating and delivering presentations [18]. Alternatively, if structured guidance is deemed necessary to meet learning outcomes, ChatGPT could be permitted selectively. For example, in the present CBL context, where consolidation and inquiry were blended, a chatbot‐free consolidation phase could be implemented, followed by a resource‐supported inquiry phase, where students fill gaps in knowledge and check understanding. Compared to blanket prohibition, this would enable the strengths of chatbots to be leveraged and may be easier to enforce. A similarly nuanced approach should be taken when developing guidelines for chatbot use, taking cultural and activity factors into account. Furthermore, health curricula should incorporate teaching on effective chatbot use and the value of cognitive effort during learning. This would reduce passive chatbot use in all contexts.

Further research is needed to quantitatively assess the impact of chatbots on conventional active learning methods. Also, the present study suggests students use chatbots most during independent revision, so qualitative research on how they affect cognitive effort in this context is needed.

### 4.4 Strengths and Limitations

A key strength was that observational data provided insights unaltered by student interpretations. Also, the study examined an authentic curricular environment, enhancing transferability. Limitations included potential power dynamics from researchers also serving as tutors and initial encouragement to use chatbots, which may have influenced behaviour.

## 5 Conclusion

AI chatbot use in conventional curricular activities can automate consolidation and higher‐order thinking, particularly when motivation is extrinsic, and tasks are highly structured. Health curricula must adapt to mitigate these risks while leveraging the value of chatbots for knowledge acquisition.

## Author Contributions

**A. W. R. Taylor:** conceptualization, investigation, writing – original draft, methodology, writing – review and editing, formal analysis, visualisation, data curation, resources, project administration, validation. **F. Y. Patel:** writing – review and editing, investigation, methodology, data curation, formal analysis, resources, validation. **E. K. Smyth:** writing – review and editing, investigation, methodology, data curation, formal analysis, resources, validation. **S. Gay:** supervision, conceptualization, writing – review and editing, validation. **T. Bird:** conceptualization, writing – review and editing, supervision, resources.

## Funding

The authors have nothing to report.

## Conflicts of Interest

The authors declare no conflicts of interest.

## Supporting information


**Appendix A.** Consent form.
**Appendix B.** Field note template.
**Appendix C.** Focus group schedule.

## Data Availability

The data that support the findings of this study are available from the corresponding author upon reasonable request.
