# Supplementary material for: AI in the Classroom: Observing Preclinical Students' Use of ChatGPT During Case‐Based Learning at a UK Medical School
Source: Clin Teach. 2026 Feb 13;23(2):e70360. doi: 10.1111/tct.70360 (PMC12904756; doi:10.1111/tct.70360)

# Appendices

Appendix A: Consent Form


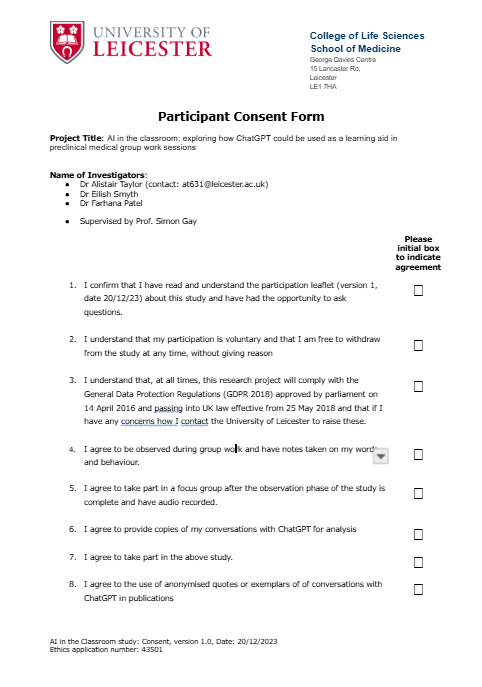


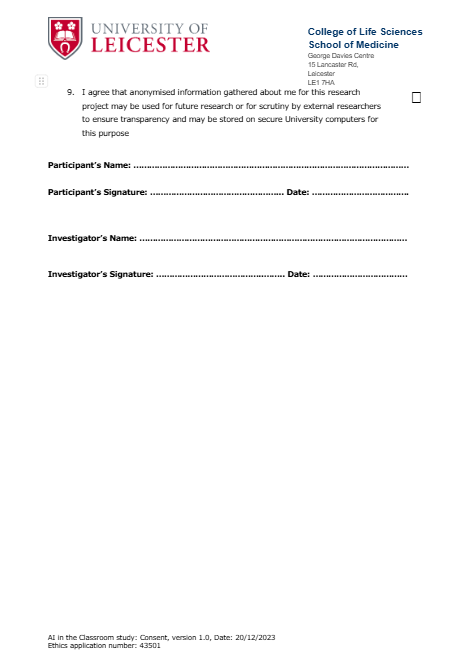


Appendix B: Field note template


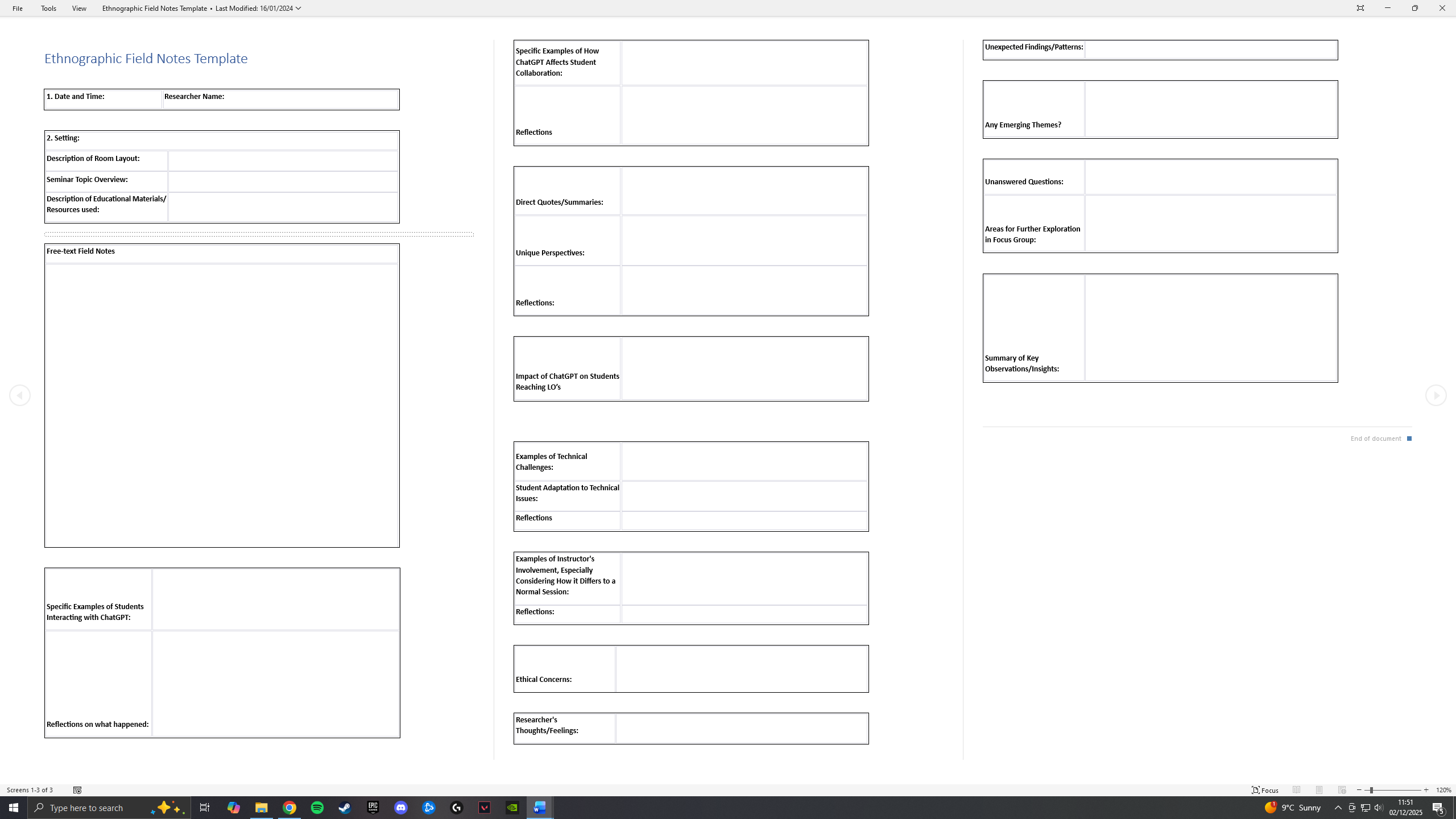


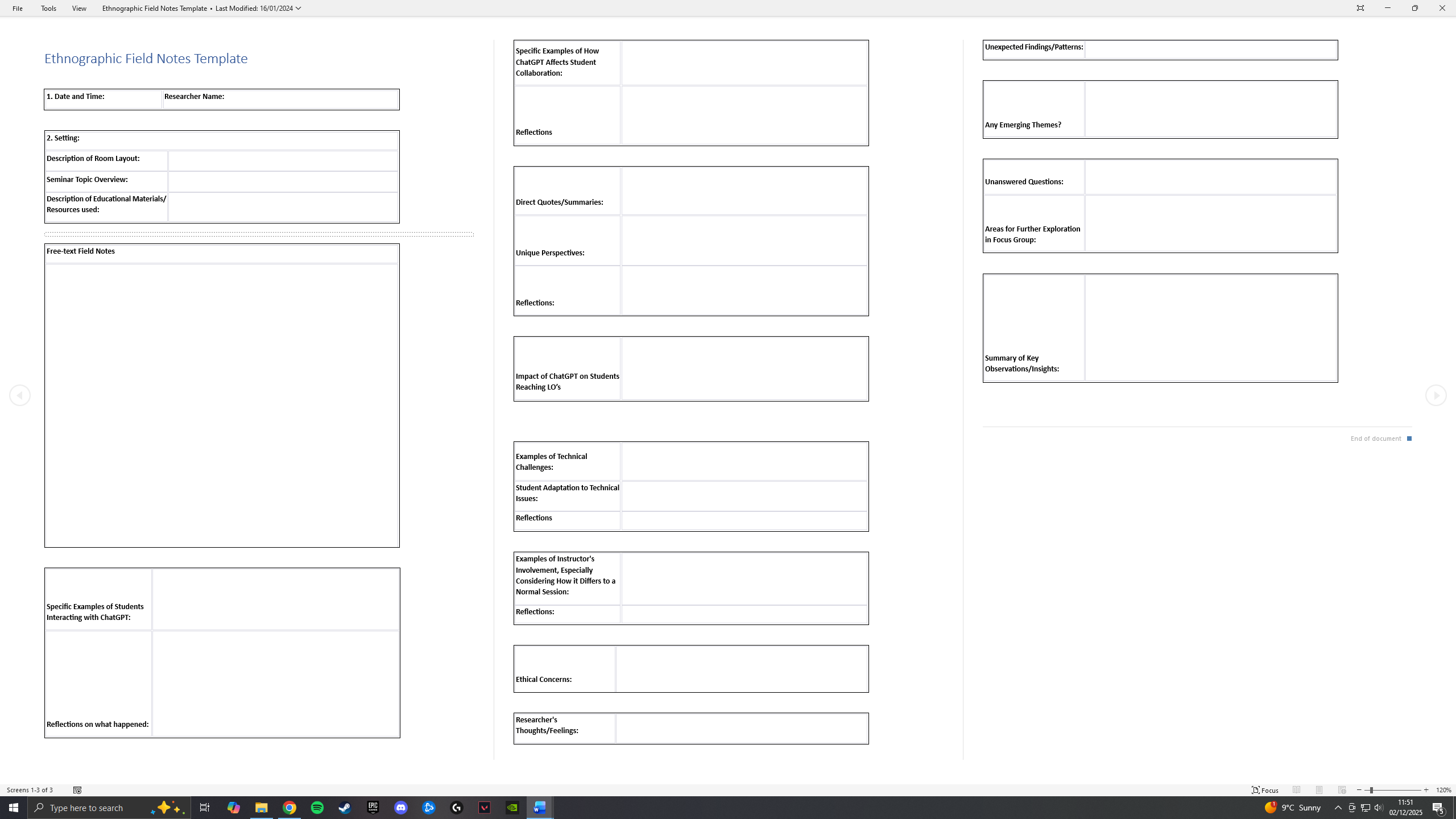


#
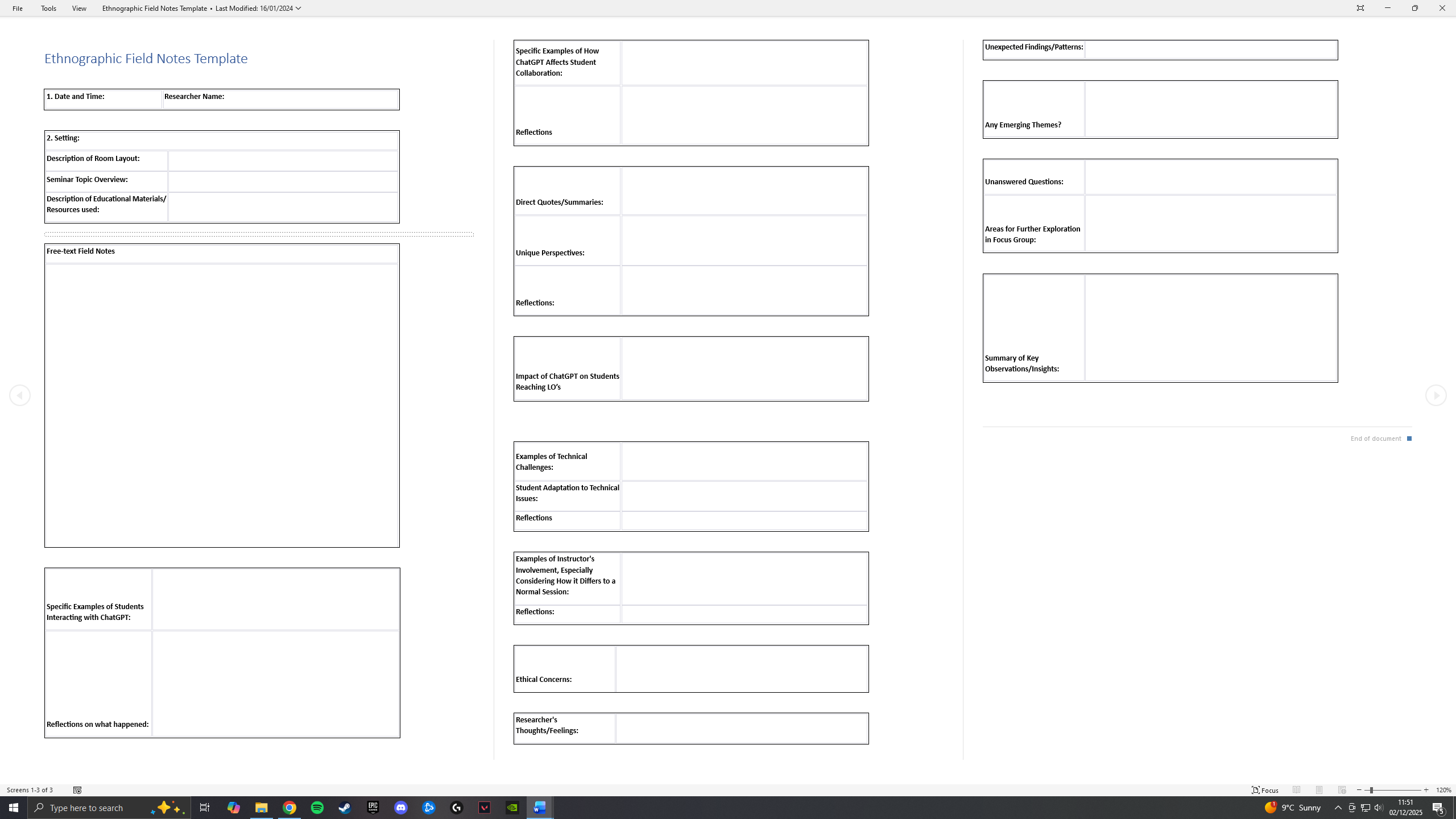


Appendix C: Focus Group Schedule


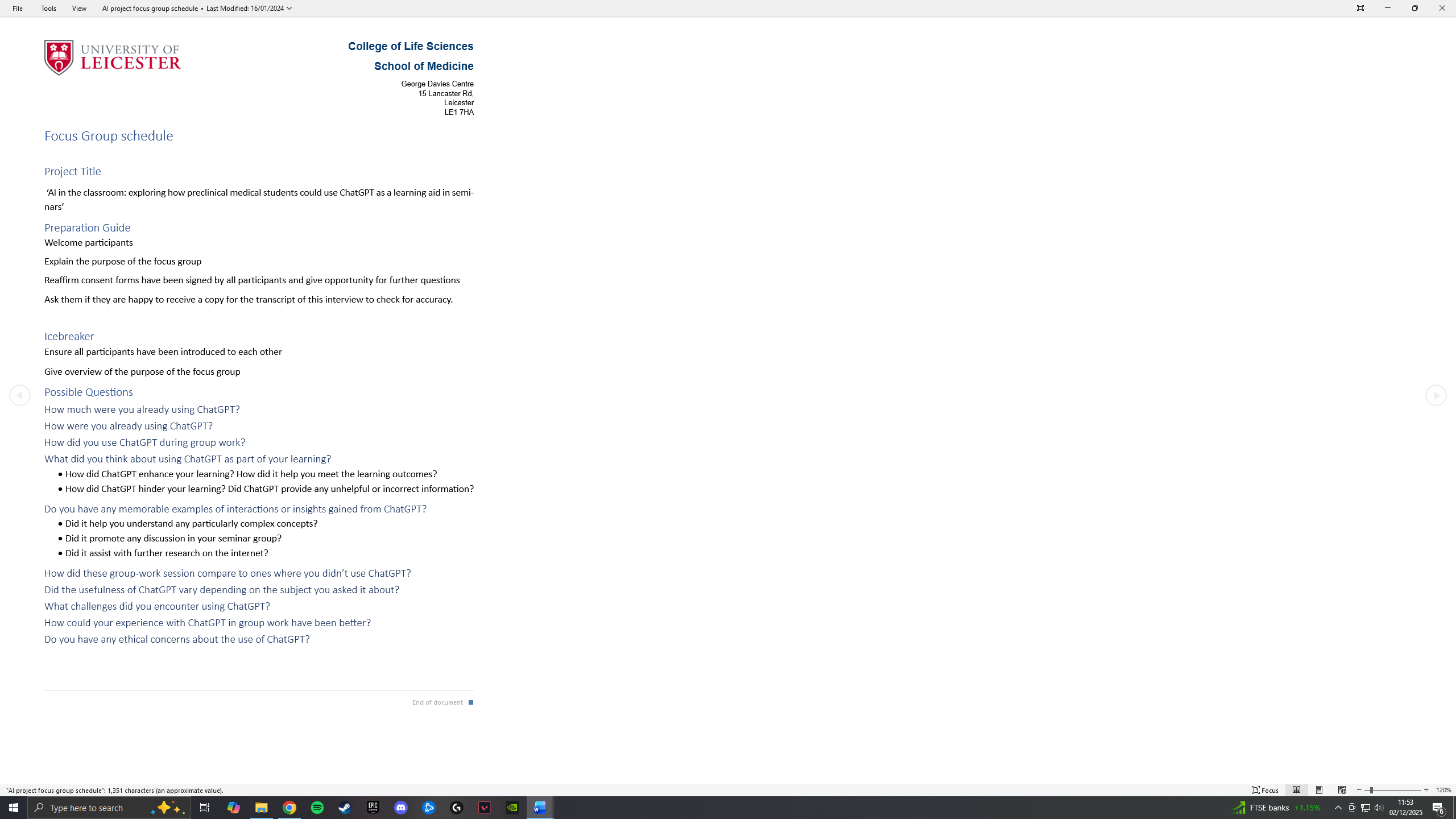

Supplement: Supplementary file 1 — Appendix A. Consent form. Appendix B. Field note template. Appendix C. Focus group schedule. [file TCT-23-e70360-s001.docx]
